# Diurnal Release of Airborne Pathogen Spores in Greenhouses via the Synergistic Effects of Relative Humidity and Wind

**DOI:** 10.1002/advs.202501500

**Published:** 2025-05-11

**Authors:** Jiayi Ma, Ali Chai, Yanxia Shi, Xuewen Xie, Lei Li, Sheng Xiang, Xianhua Sun, Tengfei Fan, Baoju Li

**Affiliations:** ^1^ State Key Laboratory of Vegetable Biobreeding Institute of Vegetables and Flowers Chinese Academy of Agricultural Sciences Beijing 100081 China

**Keywords:** airborne pathogen, greenhouse humidity, pathogen transmission, spore dispersal, target leaf spot

## Abstract

The occurrence of diseases during greenhouse vegetable cultivation is becoming increasingly severe. Humidity and wind are important factors affecting the spread of many pathogenic fungal spores, but it remains difficult to explain the phenomenon of rapid spore spread in greenhouses. Here, the detachment of spores from hyphae during rapid drops in humidity and their subsequent dispersal due to wind is detailed. It is demonstrated that *Corynespora cassiicola* spores exhibit jerking movements during humidity reduction, resulting in spore discharge, and that spore connections are weaker in high‐humidity environments than in low‐humidity environments. This investigation across the fungal kingdom further reveals that jerking movements are common in the tested hyphomycete spore species. Spores rely mainly on wind to spread after being discharged from hyphae, and their spread range is influenced by factors such as wind speed, spore source height, and spore age. In summary, it is discovered that the combined effects of diurnal humidity fluctuations and wind drive the rapid spread of pathogenic spores in greenhouses, providing a theoretical basis for optimizing control strategies for airborne fungal diseases in greenhouses.

## Graphical abstract

Day‐night cycles trigger sharp relative humidity (RH) shifts in greenhouses. Rapid humidity drops dehydrate Corynespora cassiicola spores, inducing shrinkage, jerking movements, and weakened inter‐spore connections that drive detachment. Wind is essential for dispersal, with even mild airflow enabling rapid pathogen spread. These synergistic effects of humidity dynamics and wind underscore the mechanism behind explosive greenhouse epidemics.

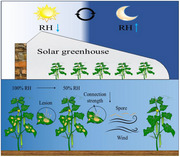

## 1 Introduction

The cultivation of vegetables in greenhouse structures is becoming increasingly common as a crop production system.^[^ ^1^ ^]^ However, the environmental conditions inside a greenhouse favor the development of microbial diseases.^[^ ^2^ ^]^ Compared with plant diseases that occur in open fields, the spread of pathogens in greenhouses is faster, and the patterns are unclear due to the semiclosed environment, leading to increasingly severe vegetable diseases.

It is widely recognized that plant pathogenic fungal spores are dispersed in a three‐stage process: active or passive release from the host, transport by biotic or abiotic factors, and eventual deposition on a new host. The occurrence and spread of plant diseases in greenhouses result from complex interactions among the host, pathogen, and environment. Environmental conditions simultaneously affect both the host and pathogen, thus playing a crucial role in disease development.^[^ ^3^, ^4^ ^]^ Specifically, humidity significantly affects pathogen spore production and release.^[^ ^5^ ^]^ For instance, Ascomycetes absorb water and swell under high humidity or submerged conditions, leading to cyst wall rupture and the subsequent ejection of ascospores.^[^ ^6^ ^]^ Basidiomycetes alter local temperature and humidity through water evaporation, creating convective air currents that remove and release spores from their surfaces.^[^ ^7^ ^]^ *Curvularia lunata* and *Helminthosporium maydis* favor spore release under low humidity conditions,^[^ ^8^ ^]^ whereas *Gibberella zeae* ascospore discharge is most effective at relative humidity levels above 92%.^[^ ^9^ ^]^ Moss spores are released mainly when the peristome teeth are opening, as the relative air humidity (RH) declines from high values to relatively low values (mainly between 90% and 75% RH).^[^ ^10^ ^]^

Wind is also a crucial factor in the dissemination of plant fungal diseases. However, particularly forceful and abrupt wind is often necessary for wind to dislodge pathogen spores. Spores that are actively ejected or dislodged by rain splash have a limited range without wind but can be transported over extensive distances when wind is present, even covering many kilometers with moderate winds.^[^ ^11^, ^12^, ^13^ ^]^ In field studies, conidia of *Alternaria dauci* were dispersed by winds above 2–3 m s^−1^ when the RH declined soon after daylight. Conidia were not detected during the night hours.^[^ ^14^ ^]^

Despite extensive research on the impact of humidity and wind on fungal spore dispersal, most studies have concentrated on spore counts rather than the cellular mechanisms underlying spore release. While environmental factors significantly influence spore dispersal dynamics, understanding the cellular mechanisms governing spore release is equally critical. These mechanisms underpin the biomechanical, molecular, and genetic processes that enable spores to transition from dormancy to active dispersal. Studying the cellular mechanisms of spore release bridges the gap between empirical observations of dispersal and the molecular logic of pathogen survival. Currently, high‐speed photography and micro/nanomechanical testing techniques make it possible to analyze the release process of spores from a host. By determining the detachment forces of the spore chain of *Aspergillus niger* via atomic force microscope (AFM) by Li et al. revealed that the average force required to detach a spore from a 4‐day‐old colony ranged from 3.27 to 4.59 nN. Similarly, the average force for a 10‐day‐old colony ranged from 1.98 to 3.05 nN.^[^ ^15^, ^16^ ^]^ Dressaire et al. observed with high‐speed cameras that evaporative cooling of the air surrounding the pileus creates convective airflows capable of carrying spores at speeds of centimeters per second. Convective cells can transport spores from gaps that may be only 1 cm high and lift spores 10 cm or more into the air.^[^ ^7^ ^]^

Cucumber (*Cucumis sativus L*.) target leaf spot (TLS), caused by the fungus *Corynespora cassiicola* (*C. cassiicola*), is a worldwide fungal disease that causes severe economic losses in cucumber‐producing areas.^[^ ^17^, ^18^ ^]^ Since 2005, 19 provinces have experienced large‐scale outbreaks of cucumber leaf spot, affecting more than 10 million acres annually and resulting in a 40–70% reduction in cucumber yield.^[^ ^19^, ^20^ ^]^ *C. cassiicola* spores are produced on target leaf spot lesions, and spore discharge occurs from humid to dry environments.^[^ ^21^, ^22^, ^23^ ^]^ The modes of transmission of TLS disease have been reported to include airborne, seedborne, soilborne, rain splash and agricultural practices.^[^ ^24^ ^]^ After successful primary infection, the plant spot produces epidermal conidia that spread to surrounding healthy plants, and the pathogen can reinfect several times during the growing season, causing the disease to become increasingly severe.^[^ ^25^, ^26^, ^27^ ^]^

In the present work, we deciphered the process by which *C. cassicola* spores detach from spore chains under changing humidity conditions and eventually disperse with the action of wind. Different spore transmittance scenarios were explored: high humidity with/without wind and low humidity with/without wind. We showed that even in the absence of wind, spores can detach from the spore chains as the humidity changes from high to low. However, wind is necessary for the spread of spores from diseased plants to adjacent plants. Using high‐speed video and depth‐sensing nanoindentation measurement techniques, we performed high‐resolution characterization of the morphology changes and connection strengths of individual spores as a function of humidity. To simulate the spread of diseases in a greenhouse environment, we detailed the effects of wind speed, spore source height, and spore age on the range of spore dispersal. These findings improve our understanding of the epidemic and transmission mechanism of airborne diseases in greenhouses and provide support for the establishment of plant disease management technologies based on greenhouse environmental regulation.

## 2 Results

### 2.1 Changes in Humidity Are Key Factors Causing the Release of *C. cassiicola* Spores

**Figure 1:**
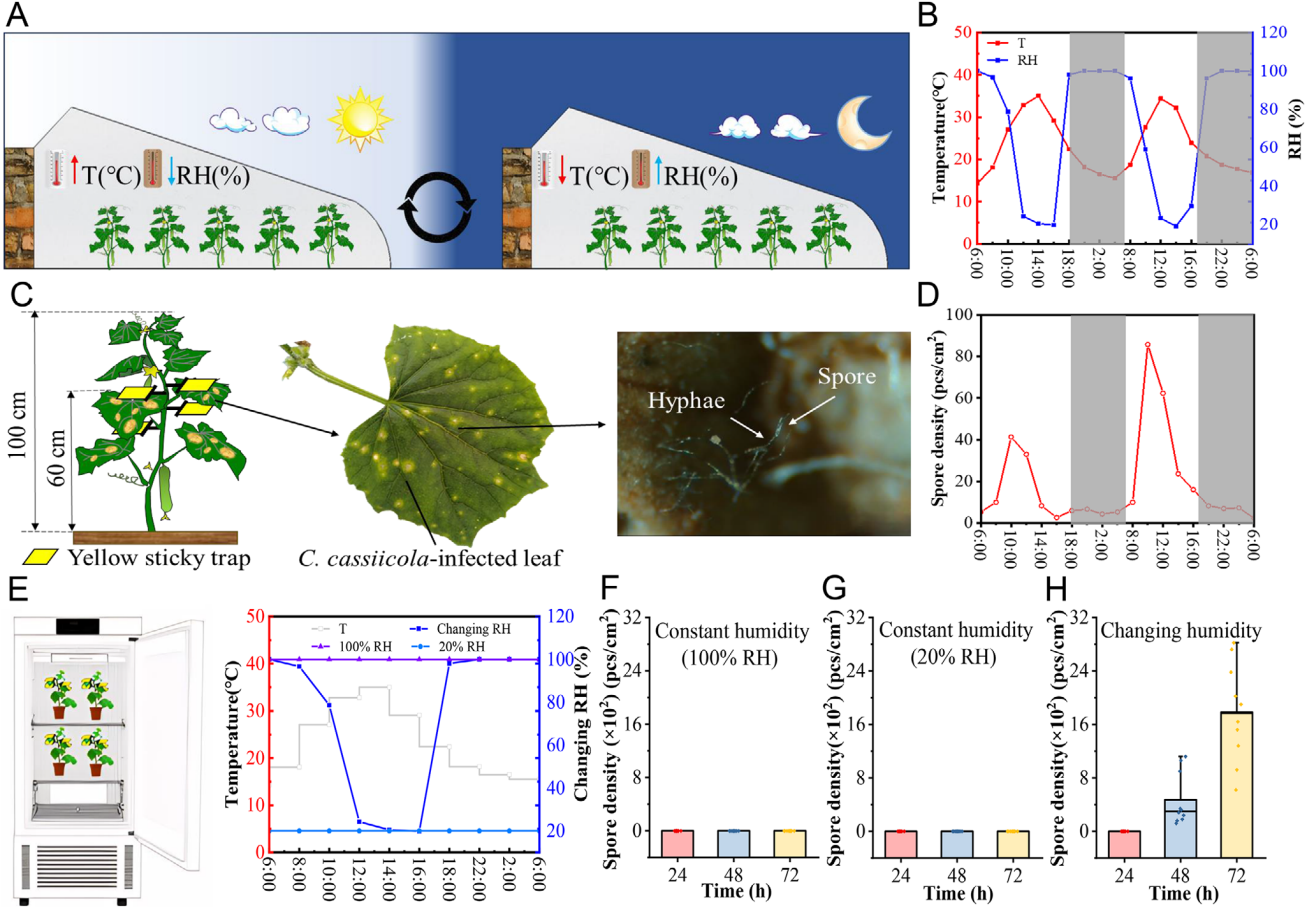
The effect of humidity on the release of spores of *C. cassiicola* under greenhouse and artificial climate chamber conditions. A) Schematic diagram of the diurnal variation patterns of temperature and humidity in greenhouse. B) Representative patterns of temperature and relative humidity changes within 24 hours in greenhouses in northern China. C) Schematic diagram of spore collection on a cucumber plant. Left: Yellow sticky traps on the underside of cucumber leaves were used to collect dropped spores. Middle: Representative *C. cassiicola*‐infected leaf; Right: Microscopic photograph of spores on the surface of cucumber leaves. D) The number of spores released from diseased leaves at different times of the day. E) Changes in temperature and humidity under constant high RH, constant low RH and changing RH. F) Number of spores released at 24, 48 and 72 h under constant 100% RH condition. G) Number of spores released at 24, 48 and 72 h under constant 20% RH condition. H) Number of spores released at 24, 48 and 72 h under changing RH condition.

*C. cassiicola* epidemics typically occur in northern China during March and April, coinciding with pronounced diurnal fluctuations in greenhouse microclimates. As shown in **Figure** **1**A,B, daytime conditions feature rising temperatures (peaking at 41 °C) and declining humidity (minimum 5%), while nighttime cooling drives rapid humidity recovery to 100%. To investigate the effect of ambient relative humidity on spore release from *C. cassiicola*, yellow sticky traps were attached to the leaves of diseased cucumber plants at 2 cm above and below the leaves via double‐headed clamps to collect and count spores after release (Figure 1C). The two variables were negatively correlated. The number of spores released by *C. cassiicola* first increased but then declined over time and was negatively correlated with relative humidity (Figure 1D). From 8:00 am to 12:00 am, the relative humidity in the greenhouse declined rapidly from 92% RH to 20% RH, and the number of spores released increased rapidly. Within 2 d, the maximum number of spores released was 41 and 86 pcs cm^−2^, respectively; during the period from 12:00 am to 16:00 pm, the relative humidity inside the greenhouse remained relatively low, below 30% RH, with small fluctuations. At this time, the number of spores released was relatively low, with the highest being 24 pcs cm^−2^ and the lowest being 3 pcs cm^−2^. From 16:00 pm to 18:00 pm, the relative humidity inside the greenhouse rapidly rose to over 90%. At this time, the number of spores released remained at a relatively low level of 3 pcs cm^−2^. During the period from 18:00 to 20:00, the relative humidity inside the greenhouse gradually increased to 100%. During the period from 18:00 pm to 6:00 am the next day, the relative humidity inside the greenhouse remained above 95% RH, and the number of spores released remained relatively low. During this two‐day period, the highest number of spores released was 7 pcs cm^−2^, and the lowest was 2 pcs cm^−2^. These data indicate that the release of spores is closely related to changes in environmental temperature and humidity. In summary, an increase in temperature and a decrease in humidity can lead to an increase in the release of spores, whereas under conditions of decreased temperature and increased humidity, the release of spores is minimal.

To clarify which temperature and humidity levels are the main factors affecting the release of *C. cassiicola* spores, spore release determination was performed in an artificial climate chamber. Cucumber plants already infected with TLS were placed under constant 100% RH, constant 20% RH and changing humidity conditions to collect the released spores (Figure 1E). The collection period was 72 h, and under constant 100% RH and constant 20% RH conditions, no spores were released within 72 h (Figure 1F,G). However, with increasing humidity, the number of spores released increased with time (Figure S3, Supporting Information). The number of spores released after 48 h and 72 h was 472 and 1782 pcs cm^−2^, respectively (Figure 1H). The released spores were not collected within 24 h, possibly due to the early onset of TLS and the inability to produce spores in the affected area. This finding indicates that both high and low humidity with constant relative humidity cannot cause spore release and that a change in relative humidity is the key factor in the release of *C. cassiicola* spores. Notably, for the abovementioned three conditions, the temperature in the chamber was set according to the variation trend in the greenhouses. Based on the spore release data in the condition of constant 100% RH, constant 20% RH, we concluded that changing temperature did not lead to the spore releasing in the constant humidity conditions.

### 2.2 Morphological Changes in *C. cassiicola* Spores with Decreasing Relative Humidity

**Figure 2:**
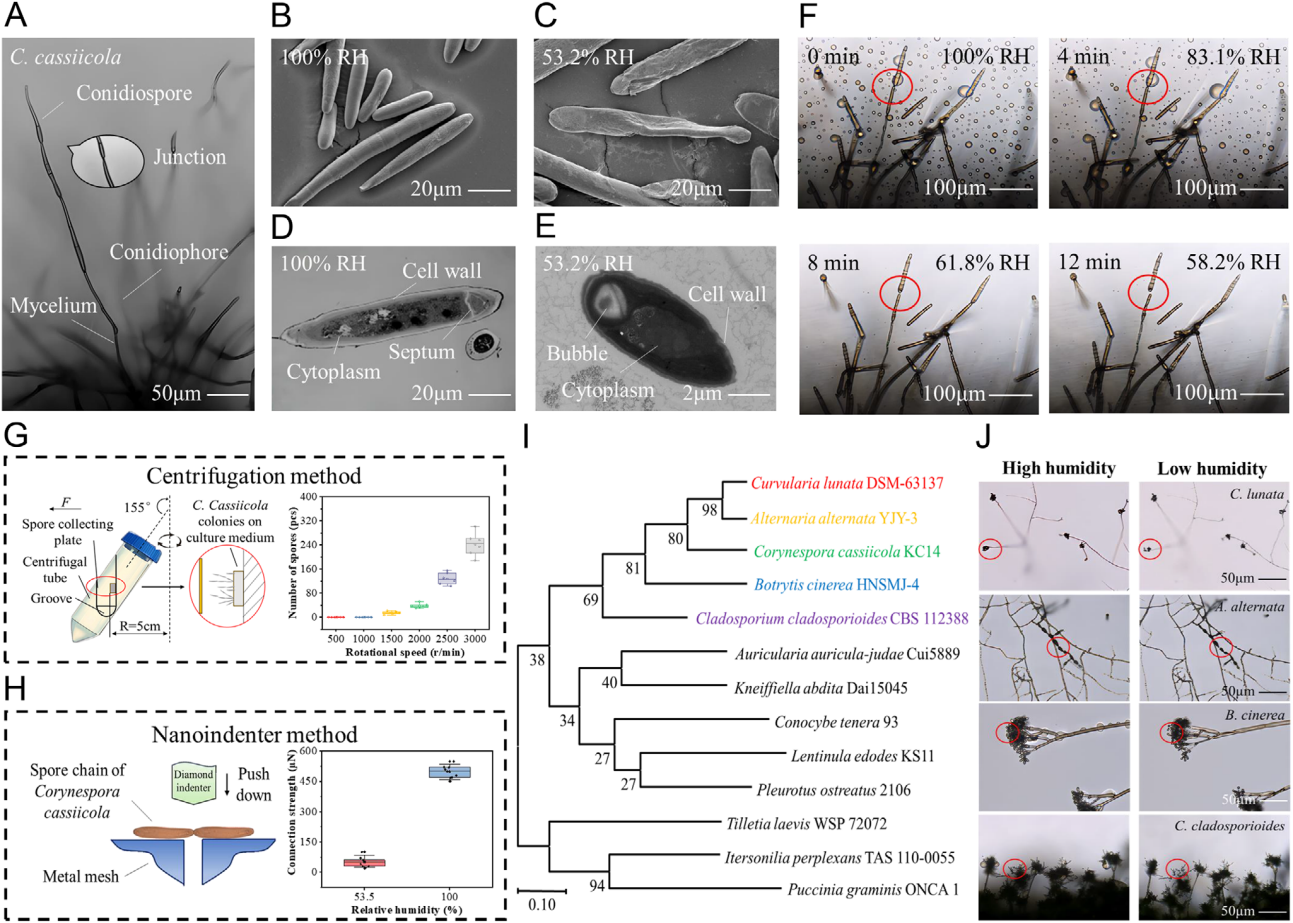
Microscopic characterization of humidity‐dependent spore release behavior of *C. cassiicola* and other fungal species. A) Representative optical microscope image of C. cassiicola. B,C) The cryo‐SEM image of *C. cassiicola* spores in B) high and C) low RH. D, E) The TEM image of *C. cassiicola* spores in D) high and E) low RH. F) *C. cassiicola* spores twist, crumple and deform with decreasing relative humidity and are eventually released and shed. G) Schematic diagram of the centrifugation method for determining the strength of connections between adjacent spores (left) and the number of released spores under different rotational speed conditions (right). H) Schematic diagram of the nanoindenter method for determining the strength of connections between spores (left) and connection strength under different loading conditions (right). I) Phylogenetic tree of fungi with different modes of spore release. J) Optical microscopy images of different types of fungi releasing spores during RH decline.

In cucumber, most *C. cassiicola* spores are in chains of 2–4 at the apex of the conidiophore, being obclavate or cylindrical, straight or slightly curved, with a truncate base and tapering apex, and containing 6–11 septa (**Figure** **2**A). Adjacent spores in the same chain are connected by a junction point that is fragile. To investigate the morphological changes in *C. cassiicola* spores with changes in relative humidity, the morphology of *C. cassiicola* spores under high and low humidity was observed by cryo‐SEM, TEM and micro‐CT. Under 100% RH, the spores exhibited an expanded state with a smooth cell wall, and the lumen was filled with cytoplasm (Figure 2B). However, at 53.2% RH, the spores exhibited a withered state with a wrinkled cell wall (Figure 2C). The inner structure of the dried *C. cassiicola* spores was further imaged via cryo‐SEM (Figure 2E) and nano‐CT (Figure S6, Supporting Information). Some gas bubbles formed in the cytoplasm (Figure 2E). This phenomenon is consistent with the gas phase hypothesis proposed by previous researchers: as spores lose water via evaporation, their volume tends to decrease.^[^ ^28^ ^]^ The spore walls, being elastic, tend to return to their original shape, but cohesion within the solution in the stipe and its adhesion to the walls oppose this tendency, and tension develops. Eventually, a point is reached when the forces of cohesion or adhesion or both are overcome, the solution breaks, and a gas phase appears. The walls are thus suddenly relieved of the inward pull and spring back to their original shape. This sudden return provides energy for spore projection.^[^ ^29^ ^]^ We hypothesize that *C. cassiicola* spores underwent similar events. To observe the movement of *C. cassiicola* spores under changing humidity conditions, we constructed an acrylic chamber in which *C. cassiicola* was cultured and observed. The humidity of the chamber could be changed on a time scale of a few minutes by adding a quantitative desiccant (Figure S4, Supporting Information). After two days of cultivation, mature spores had grown, and the RH of the chamber had reached 100%. After the desiccant was added to the chamber, the RH slowly declined from 100% to 75.5%, but the spore morphology did not change significantly. From 6–10 min, the RH continued to decline from 75.5% to 58.2%, and the hyphae and spores gradually wrinkled, twisted, and displayed a succession of jerks (Figure 2F). Some spore chains ultimately broke from the connection point (Movies S1 and S8, Supporting Information). Although gas bubble formation and the jerking behavior of *C. cassiicola* spores were mentioned in earlier studies,^[^ ^28^ ^]^ the details of spore release were not shown. In this work, the gas bubbles were clearly imaged via TEM, and the jerking movements were also video recorded, which verified the “gas phase hypothesis.”^[^ ^8^ ^]^ However, several exceptional phenomena were observed: no jolt or gas bubble formation (Movie S1, Supporting Information); jolts but without gas bubbles (Movie S2, Supporting Information); and jolts or gas bubbles but no spore release (Movie S3, Supporting Information). Thus, we believe that many other factors affect the release of *C. cassiicola* spores, such as spore age and the rate of change in humidity. For the unmatured spores, the connection strength between spore and hyphae was strong. We propose the following hypothesis: For immature spores, the linkage strength between the spore and the mycelium is relatively high, so even if vibration occurs, it cannot cause them to fall off; the cell wall of immature spores is relatively soft, and the osmotic pressure does not undergo significant changes under conditions of reduced environmental humidity, thus potentially preventing the formation of bubbles within the cells; for mature spores, a sufficiently rapid rate of humidity decrease can lead to sufficient vibration intensity, resulting in spore release.

**Figure 3:**
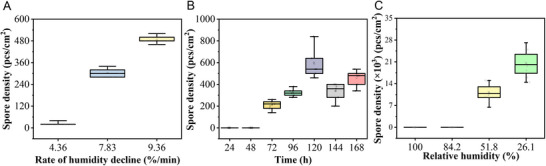
Number of released spores of *C. cassiicola* under different conditions. A) Number of released spores under different RH decline rates. *C. cassiicola* was cultured in the acrylic box at 25 °C for 72 h then different amount of calcium chloride was added to decrease the humidity in different rate (Figure S3, Supporting Information). B) Number of spores released from colonies with different ages. *C. cassiicola* was cultured in the acrylic box at 25 °C. After growing for different durations, calcium chloride was added into the box to make the RH declined from 100% to 52.5%. C) Number of released spores under different RH. *C. cassiicola* was inoculated on a 60 mm PDA medium plate and incubated at 25 °C. For the counting of released spore, the plate was placed upside down on a support platform with a yellow sticky trap. The entire platform was placed in a humidifying box. The humidity inside the humidifying box can be adjusted by adding different types of salt solutions to it (Figure S5, Supporting Information).

To determine the humidity threshold for large‐scale spore release, we designed another device that can accurately control humidity and collect spores (Figure S5a, Supporting Information). By adding various types of saturated salt solutions (disodium hydrogen phosphate, sodium chloride, ferrous chloride, magnesium nitrate and magnesium chloride) to the chamber, the RH of the chamber can be controlled at 98%, 75%, 57%, 52% and 33%, respectively. Under conditions of 100% and 84.2% relative humidity, there was no spore release. However, at relative humidities of 51.58% and 26.1%, the number of spores released was 11032 and 20266 pcs cm^−2^, respectively (Figure S5, Supporting Information). This finding indicates that the RH threshold for spore release by *C. cassiicola* is approximately 52%, and as the RH declines, the number of spores released also increases (**Figure** **3**C). This result is consistent with the response of spore morphology to humidity changes. The lower the humidity is, the more severe the spore shrinkage, the greater the deformation, and the easier it is to detach from the hyphae or spore chain.

### 2.3 The Effects of RH Decline Rate and Spore Age on the Release of Spores from *C. cassiicola*

To further quantify the effect of RH decline on *C. cassiicola* spore release, we measured the number of spores released at different RH decline rates and tested the effect of spore age on spore release at the same RH decline rate. By adding different amounts of calcium chloride to the homemade device, the rate of RH decline inside the device can be regulated. After adding 0.2 g, 0.3 g, and 0.4 g of calcium chloride, the RH decline rates in the chamber were 4.36% RH min^−1^, 7.83% RH min^−1^, and 9.36% RH min^−1^, with corresponding spore release rates of 24, 304, and 488 pcs cm^−2^, respectively (Figure 3A). This result indicates that as the rate of RH decline rate increased, the number of spores released also increased.

For the colony age‐dependent release study, the spore release dynamics of colonies of different ages were plotted at the same RH reduction rate (Figure 3B). Photographs of the *C. cassiicola* colonies at different ages are shown in Figure S2 (Supporting Information). It was found that spore release did not occur in the 24‐h‐ and 48‐h‐old colonies because those spores had not yet fully matured. For the 72 h‐old to 120 h‐old colonies, the spore release numbers were 200, 324, and 596 pcs cm^−2^, respectively. However, 144 h‐ and 168 h‐old colonies released fewer spores than 120 h‐old colonies did, with values of 312 and 440 pcs cm^−2^, respectively. It is speculated that by 120 h, spores began to aggregate between spore chains and hyphae, making it difficult for spores to detach from the colony even if they detached from the hyphae.

### 2.4 The Connection Strength between Spores under High and Low RHs

To understand the effect of RH on the connection strength between adjacent spores, we measured the binding force between *C. cassicola* spores via two methods: the nanoindenter method and the centrifugation method. The nanoindenter method was used to evaluate the critical shear force for the spore chains. *C. classicola* spores were randomly fixed on a metal mesh, as shown in Figure 2H. Two linked spores with two ends fixed at the mesh pore were chosen as test targets, and the connection point between two spores was used as the pressure point for the cantilever (Figure S9, Supporting Information). When the cantilever pressure continues to increase to a critical value, the connection point between the two spores breaks. The results revealed that the critical pressure under 100% RH conditions (497 ± 29.83 µN) was 10‐fold greater than that under 53.5% RH conditions (51 ± 23.31 µN) (Table S4, Supporting Information). These findings indicate that as the RH declines, the connection strength between spores also decreases, making it easier for spores to detach. Although many factors (spore age, fixation position, and spore shape) can affect the test results, we conducted extensive testing in each humid environment to increase the credibility of the data.

The centrifugation method is a technique that uses centrifugal force to break spore chains and evaluate the strength of connections between adjacent spores. This method is based on the principle that the adhesion between the particle and surface is thought to be equivalent to the centrifugal force generated during rotation.^[^ ^30^ ^]^ The number of detached spores versus centrifugal force was plotted (Figure 2G). Under low‐humidity conditions (55.3% RH), the spores started to detach from the colonies once the centrifugal force reached 2.83 nN at a rotational speed of 1500 rpm, whereas the critical centrifugal force rose to 11.34 nN under high‐humidity conditions (90.2% RH) (Tables S1 and S2, Supporting Information). As the centrifugal force increased, the number of detached spores also increased under both high‐ and low‐humidity conditions (Figure S8, Supporting Information). This trend was consistent with the shear force data. However, the binding force measured by the centrifugation method is much greater than that measured by the nanoindenter method, probably because the two forms of force are different—the drag force and the shear force. Shear force is a force perpendicular to the spore chain. In contrast, tension is a force parallel to the spore chain, which causes the spore to move in the direction of the applied force. These results indicated that *C. cassicola* spores were more likely to fall off the spore chain or hyphae through drag force rather than shear force. Moreover, the effect of spore age on the binding force was also considered. The binding force of colonies with different growth ages was tested via the centrifugation method. As the growth age increased, the older the colonies were, the weaker the binding force was (Table S3, Supporting Information). For the 1‐ and 2‐day‐old colonies, no spores detached at the maximum rotation rate (4000 rpm) because the 2‐day‐old colonies had not yet produced mature spores. The average binding force decreased from 2.83 to 1.27 nN as the growth age increased from 3 days to 7 days. This result was similar to previous drag force measurements on *Aspergillus niger*, which is also a hyphomycete. In *Aspergillus niger*, older colonies also presented a weaker detachment force.^[^ ^16^ ^]^

### 2.5 Spore Release Behavior among Different Fungal Kingdoms

To extend our findings, we tested spores from other clades and discovered that those from hyphomycetales, such as *Curvularia lunata, Alternaria alternata, Botrytis cinerea and Cladosporium cladosporioides*, presented similar humidity‐dependent jerking behavior (Movies S4–S7, Supporting Information). The observed variation in jerking movement among different spore species may also be related to structural variations in spore chain/spore cluster architecture (Figure 2J). For example, *C. lunata* bore a cluster of conidia at its apex; as the RH declined, conidia were commonly released while the conidiophore was stationary. The conidium slowly moved inward, outward, or to the side, as though pivoted at the point of attachment to the conidiophore, and then suddenly shot away (Movie S4, Supporting Information); Spores of Alternaria tenuis were dark, and they were produced in simple or branched chains of up to a dozen or more spores. In A. tenuis, the entire spore chain moved from side to side or twisted about itself and displayed a succession of jerks. Some of these jerks resulted in chain breakage, and single spores or groups of two or more spores were detached and thrown away, but the hyphae changed only slightly in shape (Movie S5, Supporting Information). Similar to *C. lunata* spores, spores of Botrytis cinerea deformed as RH declined and rotated slowly around the conidial peduncle attachment point for eventual release, at which point the fungus did not move except for slight deformation (Movie S6, Supporting Information). Spores of *Cladosporium cladosporioides* were similarly clustered; as the RH declined, the conidia moved outward or inward, the hyphae underwent large deformation triggering oscillation of the spore cluster, and the spores then detached from the cluster singly or in multiples (Movie S7, Supporting Information). These findings suggest that humidity‐dependent spore release is common among imperfecti. However, those from hyphomycetes (e.g., *C. cassicola* and *Botrytis cinerea*) and basidiomycetes (e.g., *Pleurotus ostreatus*, *Conocybe tenera*, and *Lentinula edodes*), as well as uredinomycetes (*Puccinia graminis*), presented different release behaviors. Most basidiomycete fungi actively eject spores. Typically, the active discharge of Auricularia auricular spores is powered by the rapid movement of a droplet of fluid, called a Buller's drop.^[^ ^31^, ^32^ ^]^ Fusion of the droplet onto the spore creates a momentum that propels the spore forward. In mushrooms, evaporative cooling of the air surrounding the mushroom pileus creates convective airflows capable of carrying spores at speeds of centimeters per second.^[^ ^7^ ^]^ Aeciospores, such as *Puccinia graminis*, can be ejected from cluster cups, which is driven by water ingress.^[^ ^33^ ^]^ Therefore, from an evolutionary perspective, humidity‐dependent jerking behavior probably evolved in the common ancestor of all the hyphomycetales.

### 2.6 Wind, in Addition to Humidity, Is a Key Factor for Spore Disposal

**Figure 4:**
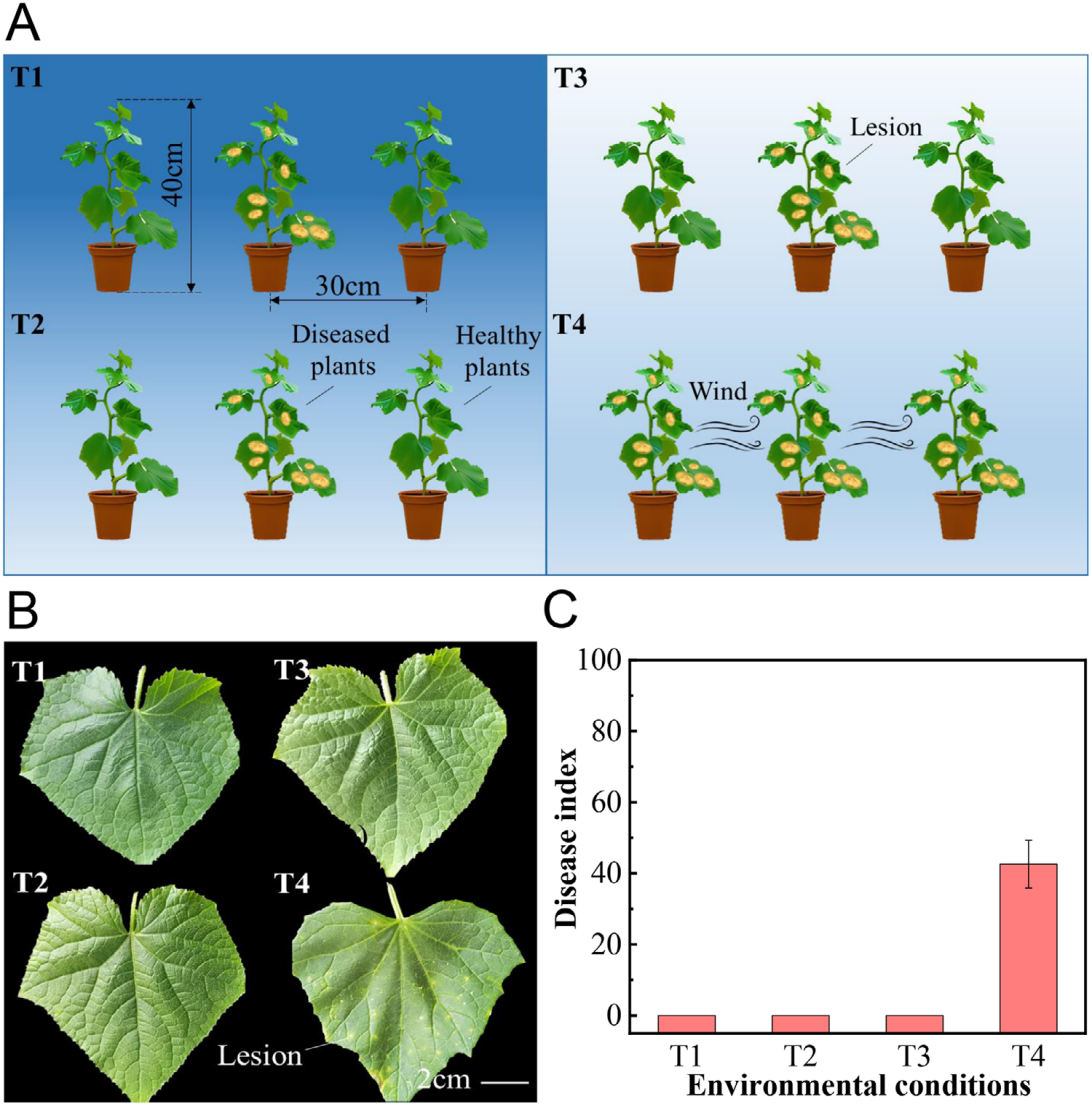
Effect of wind on the spread of cucumber TLS. A) Schematic diagram of the incidence of cucumber target leaf spot disease around cucumber plants under different environmental conditions. T1—constant 100% RH without wind, T2—constant 20% RH without wind, T3—natural high‒low RH alternation without wind and T4—natural high‒low RH alternation with wind. The TLS transmission test was performed in an artificial climate chamber. To mimic the nature condition, the RH was set as 100% at night, 20% at daytime. B) Incidence of cucumber plant leaves around infected plants under different environmental conditions. C) Disease index of cucumber plants around infected plants under different environmental conditions.

For spores that have already detached from hyphae, dispersal under the action of wind is another important procedure in the occurrence of plant diseases. To understand the range of spore dispersal, the effects of wind on spore dispersal were investigated. First, we validated that wind was the key factor for spore dispersal in cucumber plants. Four types of growth conditions were designed: constant 100% RH without wind, constant 20% RH without wind, natural humidity with and without wind (**Figure** **4**A). Only under windy conditions, the healthy cucumber plants were infected with the TLS with a disease index of 42.59 ± 6.72, indicating that spores can disperse to neighboring plants under natural humidity with wind conditions (Figure 4B,C). For the other three conditions, healthy plants were not infected. Therefore, an environment with alternating wet and dry conditions, coupled with wind, is necessary for spore disposal.

To investigate the effect of wind speed on spore dispersal, we constructed a spore dispersal model in a wind tunnel (Figure S10, Supporting Information). As the wind speed increased from 0.5 up to 1 m s^−1^ with a continuous blowing time of 10 seconds, the farthest straight line dispersal of the spores increased from 30 cm to 80 cm (Figure S11a,b, Supporting Information). Moreover, when the blowing time decreased from 10 s down to 2 s, the farthest linear dispersal distance of the spore transmission distance was reduced by 70 cm (Figure S11c, Supporting Information). When the wind speed increased to 2 m s^−1^, the spore dispersal distance increased to 40 cm (Figure S11d, Supporting Information).

**Figure 5:**
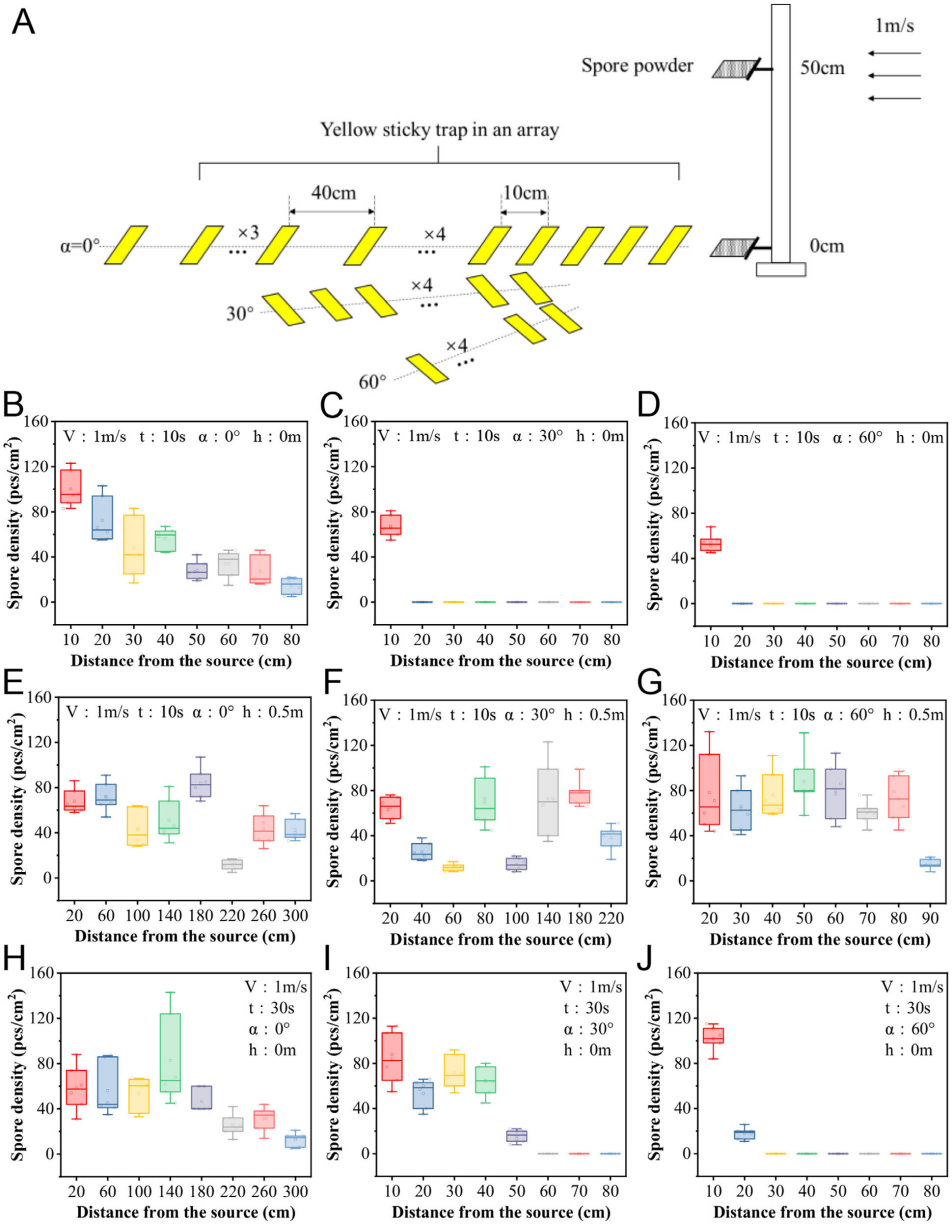
Effect of wind on the disperse of *C. cassiicola* spores. A) Experimental setup for testing spore dispersal range in wind tunnel. B–G) Dispersal distance and number of *C. cassiicola* spores in three directions (0°, 30° and 60°) under different heights (0 m and 0.5 m) of spore sources. Wind speed was set as 1 m s^−1^ with blowing time of 10 s. H–J) Dispersal distance and number of *C. cassiicola* spores in three directions (0°, 30° and 60°) under 0 m heigh. Wind speed was set as 1 m s^−1^ with blowing time of 30 s.

The effects of spore dispersal at different angles were also considered. Sticky papers were arranged in linear arrays, as shown in **Figure** **5**A. One array of papers was placed directly along the axis of the wind flow (*α* = 0°), up to a maximum distance of 3 m in front of the spore powder. Two additional linear arrays of papers were placed at *α* = 30° and 60° with respect to the wind flow. The initial wind speed was set to 1 m s^−1^ and the duration was set to 10 s. The dispersed spores were collected at different angles. In the case of *V*wind = 1.0 and spore source height of 0 m (*h* = 0 m), the farthest spore dispersal distance was 80 cm (Figure 5B); when the collection angles were 30° and 60°, the farthest spore dispersal distance was 10 cm, indicating that the straight‐line dispersal distance of spores was much greater than the dispersal distance at other angles (Figure 5C,D); when the wind duration increased to 30 s and the collection angle was 0°, the farthest spore dispersal distance was 300 cm (Figure 5H); and when the collection angle was 30° and 60°, the farthest spore dispersal distances were 50 and 20 cm, respectively (Figure 5I,J). As the wind duration increased from 10 s to 30 s, the spore dispersal distance at different angles increased to varying degrees. However, as the collection angle increased, the spore dispersal distance also decreased. These findings indicate that the duration of wind can effectively increase the spore dispersal distance, but as the offset angle increases, the spore dispersal distance effectively decreases.

Considering that the location of the lesion may be on leaves of different heights on the plant, the effect of the height of the spore source on the dispersal range was also investigated. When the height of the spore source increased to 50 cm, the farthest spore dispersal distance reached 300 cm, which far exceeded the maximum collection distance (Figure 5E). In addition, for lines with α = 30° and α = 60°, the farthest spore dispersal distances were 220 and 90 cm, respectively, which also exceeded the maximum collection distance (Figure 5F,G). As the height of the spore source increased, the dispersal distance of spores at different angles increased significantly and exceeded the maximum distance for spore collection. These findings indicate that increasing the height of the spore source can effectively increase the dispersal distance of spores. The result can be mechanistically explained airflow velocity profiles and spore sedimentation dynamics within the wind tunnel. On one hand, At higher release heights, spores are introduced into regions of faster, more uniform airflow, where reduced boundary layer friction near the tunnel floor minimizes velocity attenuation.^[^ ^34^ ^]^ On the other hand, the higher the height of the spore source, the longer the settling time, and the farther the horizontal moving distance.

**Figure 6:**
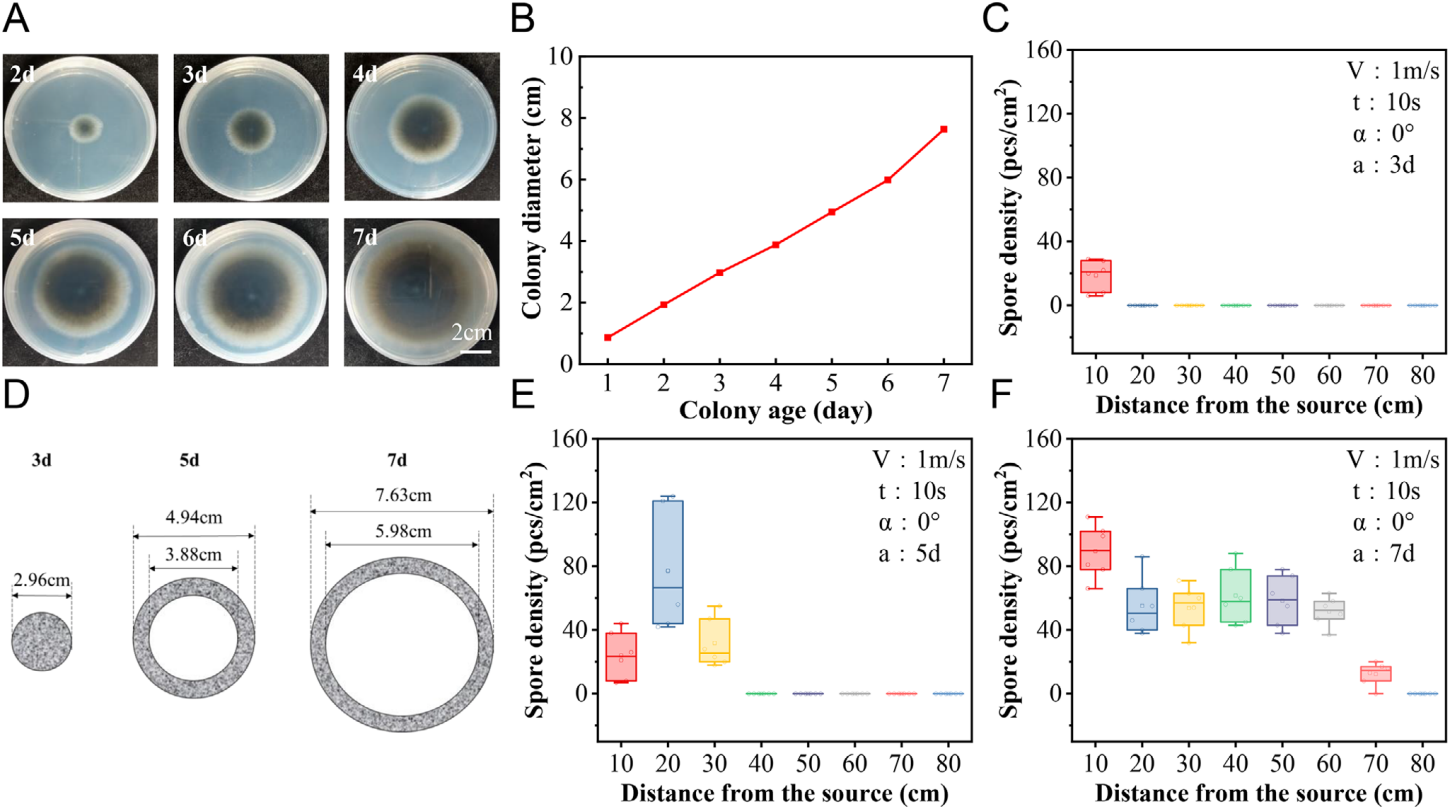
Effect of colony age of *C. cassiicola* on spore disperses behavior. A) Images of *C. cassiicola* colonies growing on PDA medium for different days. B) Changes in diameter of C. cassiicola colonies at different colony ages. C) The number of *C. cassiicola* spores dropped at different distances under the conditions of wind speed of 1 m s^−1^, blowing time of 10 s, collection angle of 0° and colony age of 3 d. D) Position of *C. cassiicola* at 3, 5 and 7 d colony age in PDA medium. E) The number of *C. cassiicola* spores dropped at different distances under the conditions of wind speed of 1 m s^−1^, blowing time of 10 s, collection angle of 0 ° and colony age of 5 d. F) The number of *C. cassiicola* spores dropped at different distances under the conditions of wind speed of 1 m s^−1^, blowing time of 10 s, collection angle of 0 ° and colony age of 7 d.

We further investigated the effect of spore age on spore dispersal behavior (**Figure** **6**A,B). For 3‐day‐old colonies, only a small number of spores were found 10 cm from the colony (Figure 6C). For 5‐day‐old colonies and 7‐day‐old colonies, both the number of released spores and the spore dispersal distance increased (Figure 6E,F). This is mainly attributed to the fact that the older the bacteria are, the weaker the connection between spores, and the easier it is to be dragged off by the wind (Figure 6D). Moreover, only a small number of spores were detected for all ages of colonies. This phenomenon can be explained by the structure of *C. cassiicola*. For spores in the same chain, the oldest spore was located at the end of the spore chain, and the septum between two older spores was generally more easily broken. Moreover, the younger spores, which were closer to the conidiophore, exhibited much stronger connections with neighboring spores or conidiophores. The minimum drag force can be considered a threshold for the detachment of spores.

## 3 Discussion

Understanding the transmission mechanism of pathogens in greenhouses is crucial for vegetable disease control. Although it was evidenced that high humidity is one of the most important determinant in crop disease outbreaks, Most previous studies only focus on the severity of disease occurrence or the number of pathogens in the environment, lacking knowledge on the cellular mechanics of pathogens, which was crucial to understanding and controlling the release of the pathogen. Here, we provide a detailed analysis of the release mechanism of *C. cassiicola* spores, which mainly includes the deformation of spores during the transition from high to low RH in the environment, leading to their detachment from the spore chain and subsequent dispersal under the action of wind. Importantly, the release of spores is not only related to the degree of RH decline but also to the rate of RH decline. The faster the RH declines, the larger spores jolt amplitude and the easier they are to fall off. We have shown that the connection force among *C. cassiicola* under high humidity conditions was much stronger than that under low humidity conditions. Furthermore, we also demonstrated that spores from the tested hyphomycetales fungal pathogen exhibit humidity‐dependent jerk and detachment behavior, which is similar to that of *C. cassiicola*. We inferred that there should be some general rules behind these phenomena. Thus, the release behavior of other hyphomycetales pathogens, especially the spore connection strength or spore chain deformation, needs to be further measured and compared in future studies. In this work, we designed unique chamber and method to control the humidity and observe the spore movement which can be used for the study in other hyphomycetales pathogens. However, the method for the spore connection strength measurement may not be suitable for other fungal species because the spore shape varied largely among different fungal species.

The observed differences in binding forces measured by centrifugation (tension) and nanoindentation (shear) reflect the anisotropic mechanical properties of *C. cassiicola* spore connections. Centrifugation applies tension parallel to the spore chain, simulating wind‐driven drag forces that dominate natural detachment scenarios. In contrast, nanoindentation induces shear perpendicular to the chain, mimicking lateral impacts (e.g., physical contact). Biologically, tension forces are more relevant to natural spore release, as wind‐driven drag aligns with the primary detachment axis. However, shear resistance data provide critical insights into spore chain robustness against incidental mechanical disturbances.

Regarding the spore dispersal, our results revealed that under windless conditions, the dispersal distance of spores does not exceed 10 cm, and spores can only spread over longer distances in the presence of wind. The effects of wind speed, wind duration, and spore height on spore dispersal distance were determined. Generally, higher wind speed, higher spore source height, longer wind blowing time could lead to larger range of spore dispersal. In theory, the dispersal distance of *C. cassiicola* spores is inherently linked to their sedimentation dynamics. Using Stokes’ law, we calculated the terminal velocity (ʋ_t_) of individual spores based on their density (153.6 kg m^−3^) and dimensions (122 µm × 7 µm):

(1) $$v_{t}=\frac{2r^{2}g\left(\rho_{\mathrm{spore}}-\rho_{\mathrm{air}}\right)}{9_{\eta }}\approx 0.12\;\mathrm{cm}\;s^{-1}$$

where *r* is the spore radius, *g* is gravitational acceleration, ρ_spore_ and ρ_air_ are spore and air densities, and η is air viscosity. This low terminal velocity enables spores to remain airborne for extended periods under minimal turbulence, facilitating long‐distance transport. However, turbulent eddies in greenhouses—often generated by ventilation or canopy interactions—may enhance localized deposition, particularly near obstacles like leaves or structural frames. Our data indicate that even low wind speeds (0.5 m s^−1^) can cause the spread of pathogens, and that the older the spores, the easier they are to detach from the hyphae. In actual greenhouse vegetable production, to meet the dual demands of disease suppression and optimal crop growth in modern greenhouses, we propose actionable strategies that integrate environmental regulation with targeted airflow management, such as timed ventilation and targeted dehumidification. First, timed ventilation protocols can minimize spore dispersal by restricting high‐speed airflow to periods of low spore release risk (e.g., nighttime or early morning when humidity remains >90%). Second, localized dehumidification systems installed near infection hotspots (e.g., diseased plant clusters) can stabilize relative humidity (RH) above the 52% threshold during daytime, reducing spore detachment without compromising overall ventilation efficiency.

While this study focused on *C. cassiicola*, our preliminary observations of other hyphomycete fungi—including *Alternaria alternata, Botrytis cinerea*, and *Cladosporium cladosporioides*—revealed analogous humidity‐dependent jerking movements during spore release (Movies S4–S7, Supporting Information). This suggests that passive detachment driven by humidity fluctuations may be a conserved mechanism among hyphomycetes, which often thrive in environments with cyclical microclimates (e.g., greenhouses). The shared reliance on spore chain deformation and weakened connections under low RH aligns with the evolutionary strategy of minimizing energy expenditure for spore dispersal in stable, high‐humidity niches. However, spore release mechanisms in other fungal clades, such as the active ejection mechanisms of Basidiomycetes (e.g., *Pleurotus ostreatus*) or the hydrostatic pressure‐driven ascospore discharge in Ascomycetes (e.g., *Gibberella zeae*), likely involve distinct biomechanical pathways. For instance, Basidiomycetes employ Buller's drop‐mediated ballistospory, which is mechanistically independent of humidity changes. These differences underscore the need for clade‐specific studies to assess the broader applicability of our findings.

### 3.1 Limitations and Field Applicability

While wind tunnel experiments provided controlled insights into spore dispersal dynamics under defined wind speeds and durations, several limitations must be acknowledged when extrapolating these results to real greenhouse environments. First, wind tunnels inherently simplify airflow patterns by assuming uniform, laminar flow, whereas greenhouses exhibit spatially heterogeneous turbulence due to structural obstructions (e.g., support frames, foliage) and temperature‐driven convective currents. For instance, plant canopies may alter spore trajectories by generating localized eddies or accelerating vertical airflow, phenomena not fully captured in our wind tunnel setup. Second, the static spore source height (0–0.5 m) in experiments does not account for dynamic spore release from leaves at varying elevations in a multi‐layered crop system. Additionally, interactions between spores and microclimate factors (e.g., transient humidity gradients near plant surfaces) were not modeled, potentially underestimating deposition rates. To bridge this gap, future studies should integrate computational fluid dynamics (CFD) simulations—calibrated with wind tunnel data—to predict spore dispersal in geometrically complex greenhouses. Field validation using IoT‐enabled spore traps and environmental sensors could further refine these models. Lastly, while our findings highlight wind speed and duration as critical dispersal drivers, operational greenhouses often employ intermittent ventilation to balance temperature and humidity, creating pulsed airflow regimes that may differentially influence spore transport. Addressing these limitations will enhance the translational value of controlled experiments for precision disease management.

Taken together, these findings contribute to the understanding of the rapid spread of airborne diseases in vegetables in greenhouses and aid in the development of ecological prevention and control strategies for greenhouse disease management through environmental regulation.

## 4 Experimental Section

### Preparation of Infected Cucumber Plants

Both the susceptible cucumber plant and the highly pathogenic *C. cassiicola* were provided by the State Key Laboratory for Vegetable Biobreeding. Cucumber lines susceptible to cucumber target leaf spot were grown under controlled growth conditions (135 µmol m^−2^ s^−1^ light for 12 h/d at 25 °C) for 6 weeks after seed germination. Healthy plants were inoculated with a suspension of *C. cassiicola* in water containing 0.1% Tween 20 as an adjuvant. The inoculated plants were then incubated in an isothermal–isohumidity chamber at 25 °C and 100% RH for 24 h, after which they were returned to the previous controlled growth conditions and humidified for 20 min each day. After 5 to 7 d, pustules of *C. cassiicola* spores had sufficiently penetrated the epidermis of the cucumber leaf.

### Spore Collection

The diseased cucumber plants were transferred to a temperature‐ and humidity‐controlled environment, and yellow sticky traps were attached horizontally to the upper and lower parts of the diseased cucumber plants via double‐headed clamps to collect fallen spores (Figure S1c, Supporting Information). The mixture was changed every two hours, and the number of spores dropped at different times was observed under a stereomicroscope. Under conditions of continuous 100% RH, continuous 20% RH and natural changes in relative humidity, the number of fallen spores on the leaves of diseased plants was determined every 24 h and statistically analyzed via the same method.

### Properties of *C. cassiicola* Spores

The shape of *C. cassiicola* spore is assumed to be a rod‐shaped cylinder, where the length and diameter were measured to be 122 ± 17.8 µm and 7 ± 2.2 µm, respectively. The cell wall thickness of *C. cassiicola* spores was measured to be 0.5 ± 0.11 µm via a cryogenic scanning electron microscope (HITACHI. Regulus 8100) at an accelerating voltage of 3 kV. When the terminal velocity of individually falling spores was measured, the density of *C. cassiicola* spores was calculated to be 153.6 ± 25.9 kg m^−3^, which is close to the density of other microspores.^[^ ^35^ ^]^

### Observation of Spore Morphology Changes

A PDA culture medium block (length × width × height 10 mm × 5 mm × 2 mm) was attached to the side of a homemade acrylic box (length × width × height 20 mm × 20 mm × 10 mm) wiped with 75% alcohol. A sterilized toothpick was used to pick out the *C. cassiicola* spores, which were subsequently inoculated onto the side of the culture medium. The temperature and humidity recording probes were attached to the bottom of the acrylic box via double‐sided adhesive. The acrylic box was sealed with a sealing film and fixed on a glass slide for cultivation at 25 °C for 48 h. Spore chains of 2–3 spores were selected for observation, the sealing film was opened, and calcium chloride was added to control the rate of RH decline. The changes in spore morphology were observed in real time under a microscope, and the temperature and humidity changes inside the acrylic box were recorded continuously via a temperature and humidity recorder (Figure S3a, Supporting Information). When the effects of different bacterial ages on spore release were measured, the culture medium inoculated with pathogenic bacteria was incubated for different durations, and the same method was used for observation and spore counting.

### Observation of Spore Dropping under Different Humidity Thresholds

*C. cassiicola* was inoculated on a 60 mm PDA medium plate and incubated at 25 °C for 72 h. Spores grew throughout the plate, and the relative humidity inside the plate reached 100%. The lid of the plate was opened, and the plate was placed upside down on a support platform with a yellow sticky trap. The entire platform was placed in a humidifying box. By pouring different saturated salt solutions into a humidifying box, different moisture balances can be maintained. The use of saturated ammonium sulfate, magnesium nitrate and magnesium chloride can maintain the relative humidity at approximately 80%, 50% and 30%, respectively (Figure S5a, Supporting Information). The temperature and humidity recording probes were placed inside the humidifying box near the flat plate, and the temperature and humidity changes inside the humidifying box were recorded in real time. The entire moisture box was sealed with a sealing film. After 24 h, the yellow sticky trap was removed from the humidifier box, and spore fall at different humidity thresholds was observed under a stereomicroscope.

### Spore Connection Strength Test by the Nanoindentation Instrument

Double‐sided adhesive tape was used to fix a 10 mm × 10 mm 150 metal mesh (pore size = 100 µm) to glass slides of the same size, which were subsequently pained with an adhesive agent (POWERBLOX Filmer‐17, The Dow Chemical Company, USA). The PDA plate with *C. cassiicola* was then quickly inverted over the metal mesh, and the bottom of the plate was gently tapped to allow the spores to fall evenly onto the metal mesh and left to stand for 1 min. The spores were firmly fixed on the metal mesh, and the sample was placed in a humidifying box with an absorbent sponge at the bottom to humidify the spores. For the connection strength measurement, the prepared sample was fixed on the substrate holder of the nanoindentation instrument G200 (KLA Corporation, USA), and the humidity of the environment was adjusted by a humidifier inside the instrument (Figure S9a, Supporting Information). The Diamond indenter used was of Berkovich type with a tip end radius of 30 nm. Two connected spores on the mesh are selected in the field of view as the test object. The position of the probe of the nanoindentation instrument was moved to the junction to measure the connection strength of the two spores(Cb, Supporting Information). The loads from 50 nN are increased until the junction broke. To minimize the error as much as possible, the indentation was performed on more than 10 samples.

### Wind Tunnel Test

Wind tunnel tests were conducted at the IEA‐II wind tunnel at the National Research Center of Intelligent Equipment for Agriculture, Beijing, China. The wind tunnel consisted of an open‐ended design with a working section of 6.0 m length, 2.0 m width, and 2.0 m height. An axial flow fan was used as the power source for the wind tunnel (Figure S10, Supporting Information). Under the combined action of the rectifier and rectifying device, a uniform and stable wind field was generated. The adjustable range of the wind speed in the working section was 0.57 m s^−1^; the turbulence was less than 0.3%, and the wind uniformity was less than 0.5%. The wind tunnel specifications fulfilled the requirements of the ISO 22856:2008 standard (ISO, 2008). The effects of different initial conditions on spore dispersal and spatial distribution were verified by adjusting factors such as the wind speed, blowing time, and height for spore source.

## Conflict of Interest

The authors declare no conflict of interest.

## Author Contributions

B.L. and T.F. designed the research; J.M. performed the research; Y.S., X.S., and A.C. analyzed data; S.X. and X.X. cultivated the cucumber plants and J.M. and T.F. wrote the paper. All authors read and approved the final manuscript and approved the version submitted for publication.

## Supporting information



Supporting Information

Supplemental Movie 1

Supplemental Movie 2

Supplemental Movie 3

Supplemental Movie 4

Supplemental Movie 5

Supplemental Movie 6

Supplemental Movie 7

Supplemental Movie 8

## Data Availability

The data that support the findings of this study are available from the corresponding author upon reasonable request.
