# Supplementary material for: Diurnal Release of Airborne Pathogen Spores in Greenhouses via the Synergistic Effects of Relative Humidity and Wind
Source: Adv Sci (Weinh). 2025 May 11;12(25):2501500. doi: 10.1002/advs.202501500 (PMC12225011; doi:10.1002/advs.202501500)
Supplement: Supplementary file 1 — Supporting Information [file ADVS-12-2501500-s009.docx]

Supporting Information

**Diurnal release of airborne pathogen spores in greenhouses via the synergistic effects of relative humidity and wind**

Jiayi Ma, Ali Chai, Yanxia Shi, Xuewen Xie, Lei Li, Sheng Xiang, Xianhua Sun, Tengfei Fan*, and Baoju Li*

Supporting Information Text

1. **Cryoelectron microscopy of *C. cassiicola* spores**

The initial *C. cassiicola* spores were transferred from cryopreservation tubes to Petri dishes containing PDA medium via a sterile toothpick and incubated at 28 °C for 7 days, or 100% RH *C. cassiicola* spore samples were subsequently processed. The spores on the samples were picked with a scalpel blade, fixed to sample holders with conductive tape and then rapidly frozen in liquid nitrogen for 30 s. The samples were transferred to the sample preparation chamber under vacuum via a cryopreparation and transfer system (Quorum PP3000T). Crushing, sublimation and gold sputtering were then performed. The samples were sublimated at -90 °C for 10 min and sputter-plated with gold for 60 s. Finally, the samples were transferred to a cryogenic sample holder (-140 °C) and observed via cryogenic scanning electron microscopy (HITACHI. Regulus 8100) at an accelerating voltage of 3 kV and different magnifications. A total of 53.2% RH *C. cassiicola* spores were obtained by drying the 100% RH *C. cassiicola* spore sample in an oven at 40 °C for 1 min and were subsequently observed via the same procedure as before.

1. **Transmission electron microscopy**

Samples of 100% RH and 53.2% RH *C. cassiicola* spores were obtained via the same method as described for the cryoelectron microscopy test. The spores were picked with a scalpel blade and fixed with a 1.5% osmium tetroxide solution for 1.5 h. After rinsing with distilled water, a 0.5% aqueous uranyl acetate solution was applied for 2 h at room temperature. After rinsing with distilled water twice, fragments of spores and mycelia were dehydrated for 15 min in a series of ethyl alcohol at subsequent concentrations of 15, 30, 50, 70, 90, 96, and 99.8% and twice in anhydrous ethanol. Dehydrated samples were embedded in Spurr Low Viscosity resin and polymerized at 60 °C for 48 h. The resin-embedded material was cut with a glass knife into half-thin sections with a thickness of 1 µm via a Reichert Ultra Cut S microtome; next, the material was contrasted with osmium tetroxide and mounted in Eukit. In turn, ultrathin sections of spores with a thickness of 75 nm were stained with an 8% solution of uranyl acetate in 0.5% acetic acid for 40 min. After rinsing with distilled water twice for 5 min, Reynolds reagent was applied for 15 min. The sections were rinsed with water again (twice for 5 min) and dried. Finally, observations of the structure of *C. cassiicola* spores were made via TEM (JEOL 100-S TEM model at 100 kV).

1. **Nano-CT microscopy**

The Petri dishes containing *C. cassiicola* spores were left uncovered and dried in an oven at 30 °C for 30 seconds. They were then inverted onto 10 × 5 cm transparent adhesive tape, with the adhesive side of the tape facing upward. The spores were dislodged and attached to the tape by tapping the bottom of the Petri dish. The tape was folded and affixed along its length. The samples were then cut into 1 × 3 mm samples, which were observed under a light microscope. Those samples displaying optimal spore morphology were selected and fixed with fixing clips. These clips were then mounted on a nano-CT for observation and three-dimensional reconstruction of their microstructures.

1. **Measurement of the binding forces of fungal spores**

The initial *C. cassiicola* strains were transferred to a test tube containing 10 mL of sterile water. The mixture was mixed with a vortex mixer (MXS, DLAB Scientific, China) for 10 min and then filtered to remove the mycelium. Next, the filtrate was diluted to a concentration of 1.14 × 10^5^ spores/mL, as determined by a hemocytometer. Five microliters of the *C. cassiicola* suspension was extracted with a pipette (UCHEN, China) and transferred to a 90 mm culture plate containing 10 mL of PDA medium. The culture plate with the *C. cassiicola* suspension on the agar was incubated in a light-free incubator (SPX-70; Zhongji Environmental Protection Technology, China) at 95% relative humidity and 21% oxygen content. The interior temperature inside the incubator was maintained at 27 °C, the optimal temperature for *C. cassiicola* growth. After 144 h of incubation, the *C. cassiicola* samples were removed for testing.

The *C. cassiicola* samples were perforated with an 8 mm hole punch according to the daily colony growth size, the 3D-printed fixation frame was placed into a 50 mL centrifuge tube, the perforated *C. cassiicola* samples were fixed on the inner side of the fixation frame by UV adhesive and irradiated by UV light for 10 s, and a 5 × 5 cm yellow sticky trap was pasted on the outer side of the fixation frame for the collection of the dislodged *C. cassiicola* spores between the *C. cassiicola* samples and the yellow sticky trap. The distance between the *C. cassiicola* sample and the yellow sticky trap was 10 mm.

If a *c* spore is approximated as a sphere, the centrifugal force imposed on a single spore in a centrifugal separation can be calculated as:

$F=m\omega^{3}R$ [1]

where *F* is the centrifugal force, N; *m* is the quantity of the *C. cassiicola* spore, which was measured to be 2.32 ng; *ω* is the angular speed, rad/s; and *R* is the distance between the *C. cassiicola* spores and the rotational axis, which was 5 cm in this investigation. The binding forces of the fungal spores with the colonies can be treated as equal to the centrifugal separation force for constant rotation.

During centrifugal separation, both single spores and spore chains can be detached. Distal spores with lower binding forces are more likely to detach during rotation and produce segregated particles. Spore chain detachment may result from centrifugal and noncentrifugal forces such as extrusion and friction. However, the influence of friction and crushing on the detachment of inner spores could not be quantitatively assessed in the present study. Therefore, only distal spores are discussed below.

The detachment ratio of the distal spores can be calculated as:

$\psi_{\det}=\frac{N_{\det}}{N_{\mathrm{all}}}$ [2]

where $\psi_{\det}$ is the detachment ratio, *N*_det_ is the number of segregated spores, and *N*_all_ is the total number of spores. After centrifugation, the spores on the collection plate were observed under a stereomicroscope.

The *C. cassiicola* colonies from different growth periods were placed in a centrifuge (LYNX 6000, Thermo Fisher Scientific, USA) for centrifugal separation for 1 min. A temperature of 25 °C and 50% RH were maintained inside the centrifuge. The parallel colonies were separated at 6 different rotation speeds ranging from 500 rpm to 3000 rpm. Parallel tests were repeated three times at each rotating speed, and the average detachment ratio was obtained. Finally, the relationship between the colony age and the centrifugal force, i.e., the binding force, was determined.

By adding 1 mL of water or 0.5 g of calcium chloride into the groove at the bottom of the 3D support frame and sealing it for 30 min, the relative humidity in the centrifuge tubes could be maintained at 90.2% RH and 44.3% RH. At this time, samples of the same colony age were centrifuged at different rotational speeds, and each experiment was repeated three times. The relationship between the relative humidity and the centrifugal force was determined.

1. **Phylogenetic tree establishment**

The ITS sequences of *C. cassiicola* and other fungi whose release mechanism has already been reported were collected from NCBI. The phylogenetic tree was constructed via the maximum likelihood method in MEGA11. Bootstrap values were from 1000 replications, and evolutionary distances were estimated in units of the number of amino acid substitutions per site, with a scale bar indicating 0.1 units. The numbers at the nodes are bootstrap values.

1. **TLS transmission test**

When the spore suspensions were prepared, 5 mm agar discs were picked and incubated for 15 days at 28 °C in darkness on PDA plates. Sterilized water with 0.03% Tween 20 was added to each agar plate, and cultures of *C. cassiicola* were washed from the agar surfaces by gently scratching them with a sterilized soft brush. The resulting suspension was filtered through four layers of sterile gauze into 50 mL conical tubes. The final *C. cassiicola* spore suspension was adjusted to 1 × 10^5^ spores/mL via a hemocytometer for inoculation experiments.

Cucumber seedlings with two leaves were inoculated at six points with a micropipette using 10 mL of *C. cassiicola* spore suspension (1 × 10^5^ spores/mL) on the adaxial side of each true leaf. Healthy cucumber seedlings inoculated with sterile water served as the control. These infected plants were then incubated at 28 ± 2 °C and 95 ± 5% RH in the climatic chamber. The TLS symptom can be observed after 5 days of incubation.

TLS transmission test was performed in a climate chamber. Four environmental conditions were set up to verify the importance of wind in the dispersal of *C. cassiicola* spores: T1—constant 100% RH without wind, T2—constant 50% RH without wind, T3—natural high‒low RH alternation without wind and T4—natural high‒low RH alternation with wind (1 m s^-1^). Five diseased plants were lined up in a row, and five healthy cucumber plants were placed on the left and right sides at a distance of 30 cm each, also spaced 10 cm apart. The diseased plants from the different treatments were removed after 24 h of incubation, and the remaining plants were incubated at 28 ± 2 °C and 95 ± 5% RH for 7 days to investigate the disease index of the cucumber plants. The entire experiment was repeated 3 times. Cucumber target spot disease leaf grading criteria:

Grade 0: no spots on the leaf blade, normal plant growth;

Grade 1: the lesion area accounts for less than 5% of the whole leaf area;

Grade 3: the lesion area accounts for 6%~10% of the whole leaf area;

Grade 5: the lesion area accounts for 11% to 25% of the entire leaf area;

Grade 7: 26%~50% of the leaf area of the lesions;

Grade 9: the lesion area accounts for 50% of the entire leaf area.

Disease index = [∑(incidence grade × number of diseased leaves corresponding to grade)/(total number of surveyed leaves × highest grade of surveyed incidence)] × 100


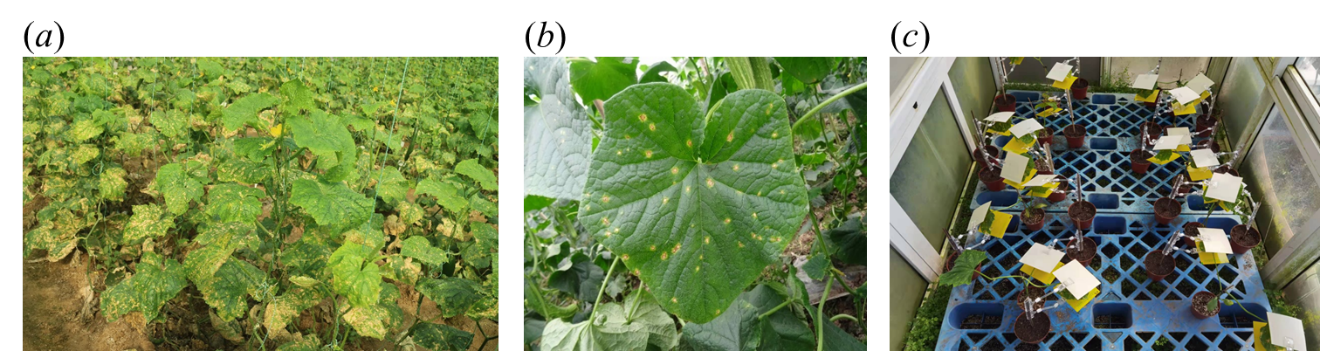


**Figure S1.** *a*) Cucumber target leaf spot disease in the greenhouse. *b*) Symptoms of cucumber target spot disease on cucumber leaves. *c*) The yellow sticky traps were fixed to the upper and lower surfaces of the infected cucumber leaves using double-headed clamps for the purpose of spore collection.

**Figure S2.** Microscopic photograph of the growth status of *C. cassiicola* spores after inoculation at different time periods. *a, b, c)* 24, 48, 72 hours of the growth status of *C. cassiicola* spores.

**Figure S3.** Microscopic photograph of yellow sticky trap used for continuous collection of *C. cassiicola* spores at different humidity settings. ImageJ were used to separate the spores (white dots) from the background. Spore drop of *C. cassiicola* spores under different humidity variations at different times of the interval. The yellow sticky traps are affixed to the upper and lower surfaces of the infected cucumber leaves using double-headed clamps. *a, d, e*) 24, 48, 72 hours of continuous collection under constant 20% RH. *b, e, h*) 24, 48, 72 hours of continuous collection under constant 100% RH. *c, f, i*) 24, 48, 72 hours of continuous collection under high-low RH alternation condition.

**
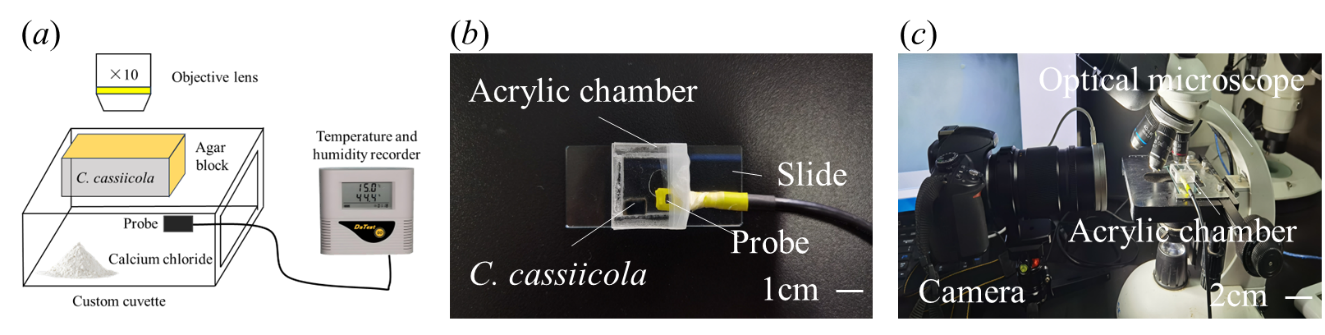
**

**Figure S4.** (*a*) Schematic of the observation device for the release of *C. cassiicola* spores in response to changes in relative humidity. (*b*) Self-constructed device for observing the release of *C. cassiicola* spores in response to a decrease in humidity. (*c*) Camera with macro lens captures the release of *C. cassiicola* spores as relative humidity decreases.

**Figure S5.** Testing the number of spores released from bacterial colonies under different relative humidity. *a*) Schematic of the device for determining the RH threshold for the release of *C. cassiicola* spores. *b-e*) Microscopic photograph of yellow sticky trap used for collection of *C. cassiicola* spores at 100% RH b), 84.2% RH *c*), 51.8% RH *d*), 26.1% RH *e*). ImageJ were used to separate the spores (white dots) from the background.


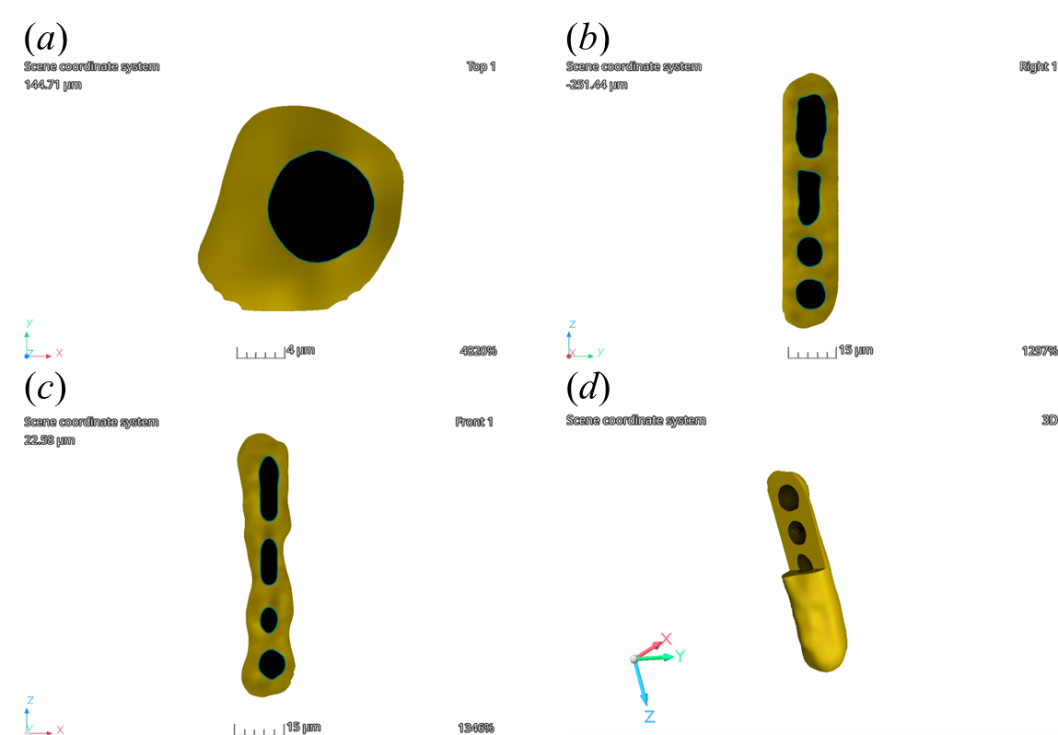


**Figure S6.** Nano-CT microscopy of *C. cassiicola* spores. *a*) Main view of *C. cassiicola* (sectional drawing). *b*) Top view of *C. cassiicola* (sectional drawing). *c*) Side view of *C. cassiicola* (sectional drawing). *d*) 3D view of *C. cassiicola* (partial sectional drawing).


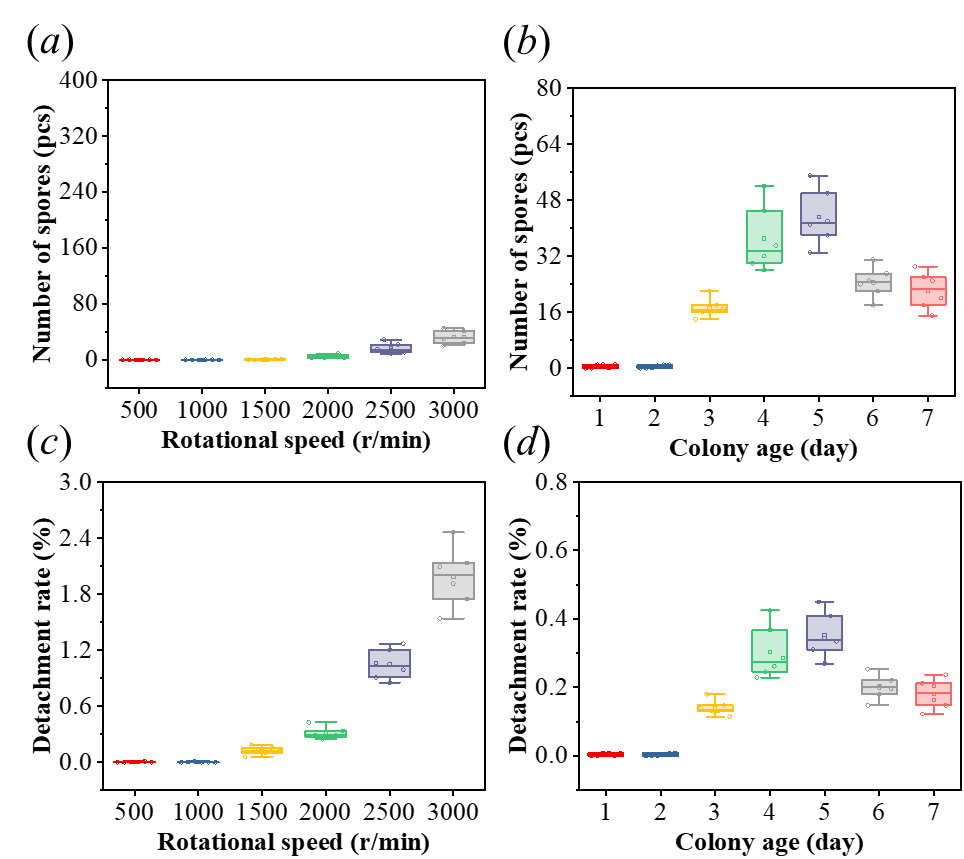


Figure S7. *a*) Number of detached *C. cassiicola* spores at different rotational speed under 3-day-old colony conditions. *b*) Number of detached *C. cassiicola* spores under different colony age at 1500 r/min. *c*) Detachment rate of *C. cassiicola* spores at different rotational speeds. *d*) Detachment rate of *C. cassiicola* spores at different colony age.


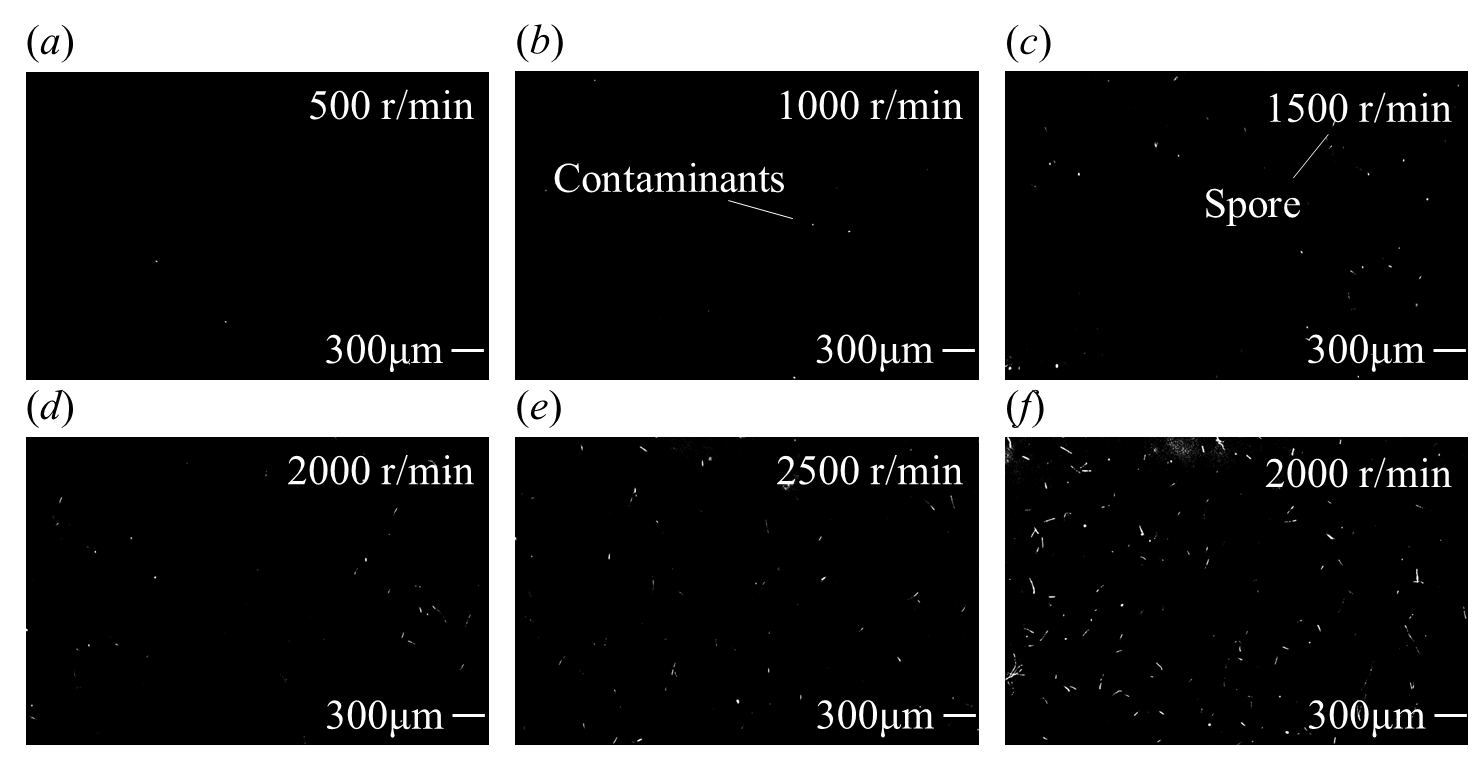


**Figure S8.** Microscopic photograph of *C. cassiicola* spores dropped at different rotational speed. 3-day-old colonies were used for this test. *a*) Rotation speed: 500 r/min. *b*) Rotation speed: 1000 r/min. *c*) Rotation speed: 1500 r/min. *d*) Rotation speed: 2000 r/min. *e*) Rotation speed: 2500 r/min. *f*) Rotation speed: 3000 r/min. ImageJ were used to separate the spores (white dots) from the background.


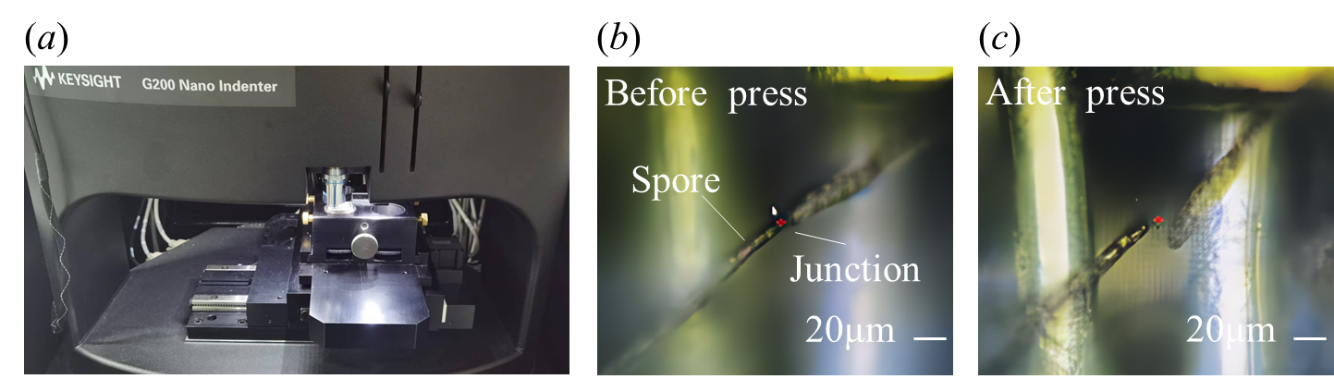


**Figure S9.** *a*) Determination of the connection strength of *C. cassiicola* spores by the G200 nano indenter. *b*) State of high relative humidity *C. cassiicola* spore chains before addition of 500 µN loads. *c*) State of high relative humidity *C. cassiicola* spore chains after addition of 500 µN loads.


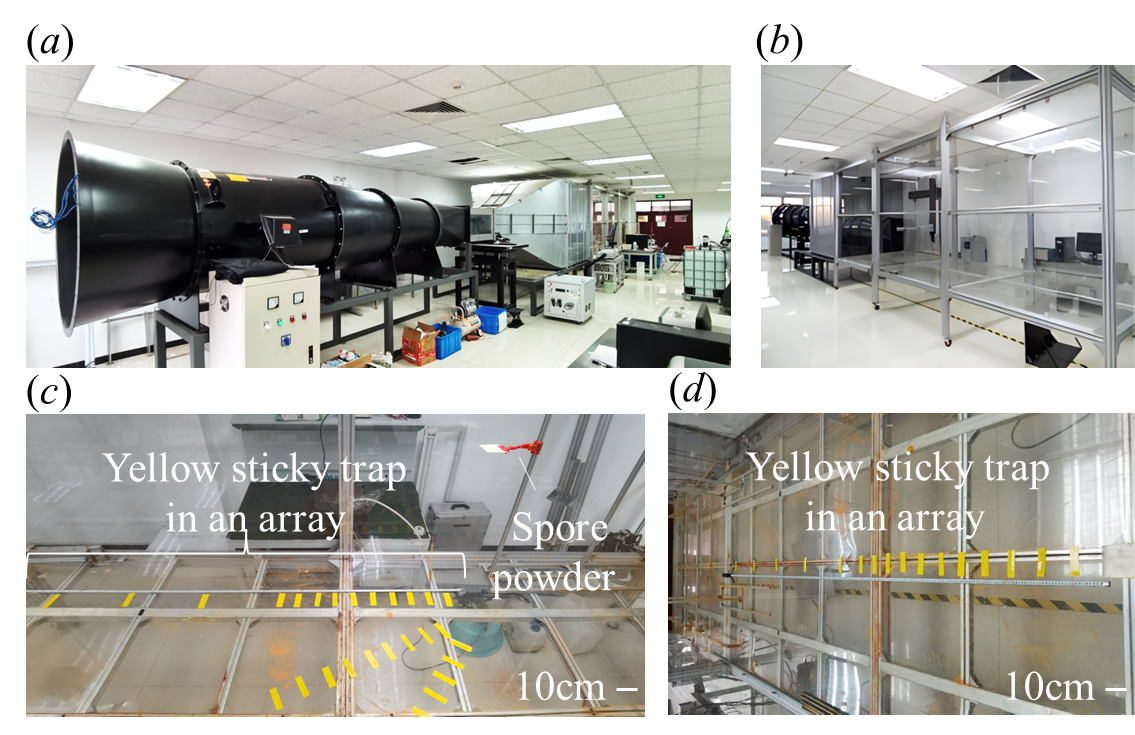


**Figure S10.** *a*) IEA-II wind tunnel test platform at the National Research Center of Intelligent Equipment for Agriculture. (*b*) IEA-II wind tunnel test platform test section. *c*) *C. cassiicola* spores were tested for distance travelled at different source heights and were collected by laying yellow sticky boards at 0°, 30° and 60°. *d*) Tests of *C. cassiicola* spores spreading distance under different wind speed conditions.


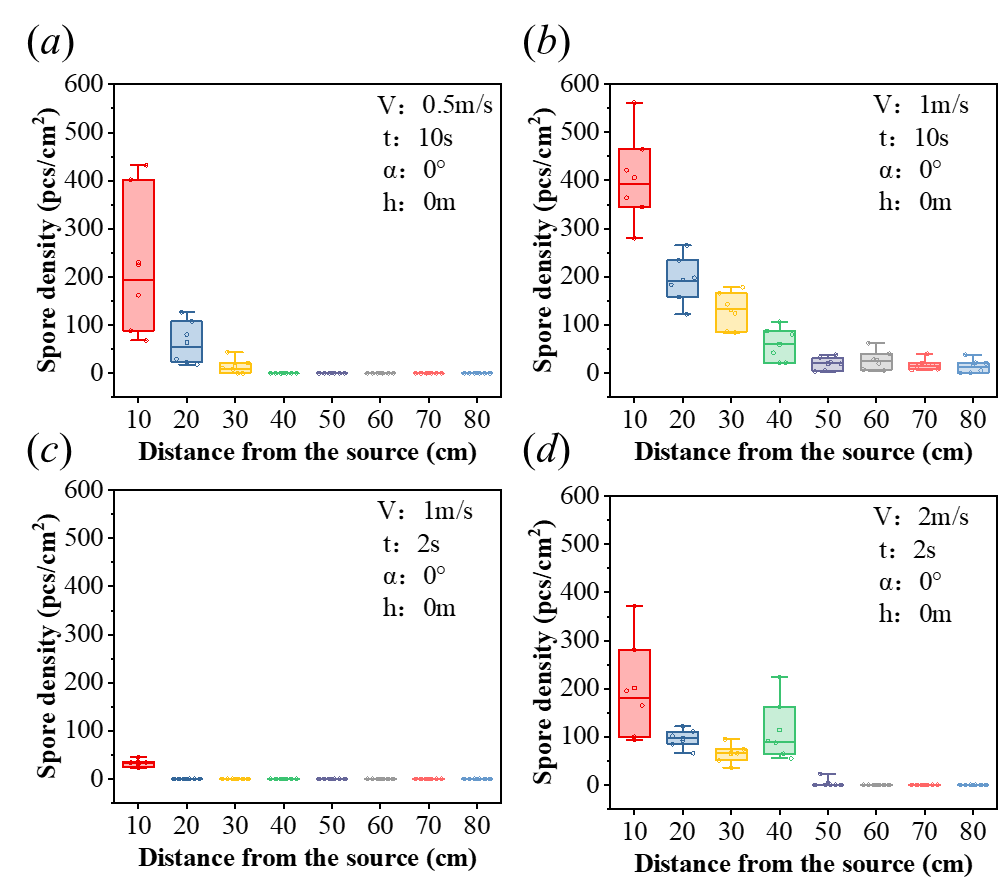


**Figure S11.** Density of *C. cassiicola* spores collected at different distances from the source of the source with different wind speeds and wind blowing times. *a*) Velocity: 0.5 m s^-1^ time: 2 s angle: 0 °heights: 0 m；*b*) Velocity: 1 m s^-1^ time: 10 s angle: 0 °heights: 0 m；*c*) Velocity: 1 m s^-1^ time: 2 s angle: 0 °heights: 0 m；*d*) Velocity: 2 m s^-1^ time: 2 s angle: 0 °heights: 0 m**.**


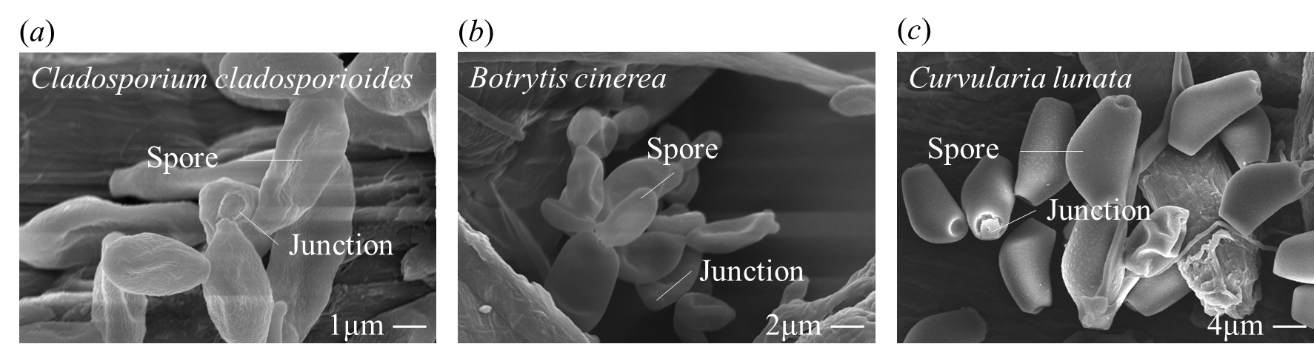


**Figure S12.** Scanning electron microscopic images of different fungal spores and junctions. *a*) *Cladosporium cladosporioides* spores. (*b*) *Botrytis cinerea* spores. *c*) *Curvularia lunata* spores*.*

Table S1. Number of *C. cassiicola* spores released at different rotational speeds for 3-days colony age at 53.2% RH.

| Rotation speed  (r/min)  N(pcs) | 500 | 1000 | 1500 | 2000 | 2500 | 3000 |
| --- | --- | --- | --- | --- | --- | --- |
| Dropped  *C. cassiicola*  spores | 0 | 0 | 12 | 33 | 147 | 234 |
|  | 0 | 0 | 16 | 30 | 130 | 301 |
|  | 0 | 0 | 18 | 35 | 104 | 188 |
|  | 0 | 0 | 22 | 35 | 155 | 256 |
|  | 0 | 0 | 14 | 41 | 111 | 214 |
|  | 0 | 0 | 7 | 52 | 121 | 261 |

Table S2. Number of *C. cassiicola* spores released at different rotational speeds for 3-days colony age at 90.1% RH.

| Rotation speed  (r/min)  N(pcs) | 500 | 1000 | 1500 | 2000 | 2500 | 3000 |
| --- | --- | --- | --- | --- | --- | --- |
| Dropped  *C. cassiicola*  spores | 0 | 0 | 0 | 5 | 22 | 33 |
|  | 0 | 0 | 0 | 3 | 14 | 30 |
|  | 0 | 0 | 1 | 9 | 29 | 41 |
|  | 0 | 0 | 0 | 7 | 15 | 45 |
|  | 0 | 0 | 1 | 3 | 11 | 24 |
|  | 0 | 0 | 0 | 3 | 9 | 21 |

Table S3. Number of *C. cassiicola* spores released by different colony ages-dependent on 53.2% RH at 1500 r min^-1^.

| Colony age  (day)  N(pcs) | 1 | 2 | 3 | 4 | 5 | 6 | 7 |
| --- | --- | --- | --- | --- | --- | --- | --- |
| Dropped  *C. cassiicola*  spores | 1 | 1 | 16 | 32 | 42 | 22 | 25 |
|  | 1 | 0 | 16 | 30 | 50 | 25 | 26 |
|  | 0 | 1 | 18 | 35 | 38 | 31 | 20 |
|  | 0 | 0 | 22 | 45 | 33 | 18 | 15 |
|  | 1 | 1 | 14 | 28 | 55 | 27 | 29 |
|  | 0 | 0 | 17 | 52 | 41 | 24 | 18 |

Table S4. Applied loads during spore chain breakage of *C. cassiicola* spores under different relative humidity conditions.

| RH  (%)   Strength(μN) | 53.5 | 100 |
| --- | --- | --- |
| Load applied | 50 | 500 |
|  | 20 | 470 |
|  | 30 | 450 |
|  | 60 | 550 |
|  | 100 | 500 |
|  | 50 | 520 |
|  | 70 | 480 |
|  | 60 | 470 |
|  | 40 | 510 |
|  | 30 | 520 |

Movie legends.

Movie S1： As the relative humidity decreased, *C. cassiicola* spore chains contracted, twisted and eventually spores were released, some spores were deformed but not released.

Movie S2： As the relative humidity decreased, *C. cassiicola* spores were concavely deformed and oscillated considerably, but the spores eventually failed to be released and no air bubbles were produced inside.

Movie S3： As the relative humidity decreased, C. cassiicola spores underwent slight deformation and small oscillations, and visible air bubbles were produced inside the spores but eventually failed to be released

Movie S4： *Curvularia lunata* spore release with decreasing relative humidity.

Movie S5： *Alternaria alternata* spore release with decreasing relative humidity.

Movie S6： *Botrytis cinerea* spore release with decreasing relative humidity.

Movie S7： *Cladosporium cladosporioides* spore release with decreasing relative humidity.

Movie S8： As the relative humidity decreased from 100% RH to 57.5% RH, *C. cassiicola* spores fell off in large numbers, and the spores in the field of view were observed from none to some, and the spores in the field of view where no change in the relative humidity occurred were mature and fell off by themselves.
